# Tumor-Infiltrating Lymphocytes Demonstrate Distinct Behavior in the Tumoral and Peritumoral Microenvironment After Neoadjuvant Chemotherapy in Patients with Resected Adenocarcinoma of the Gastric or Gastroesophageal Junction: Results from a Single Center

**DOI:** 10.3390/cancers17121971

**Published:** 2025-06-13

**Authors:** Fotios Seretis, Antonia Panagaki, Sofia Ritsatou, Eleni Stoupi, Andreas Theodorou, Spyridon Smparounis, Chrysoula Glava, Maria Theochari, Tania Triantafyllou

**Affiliations:** 1 First Propaedeutic Department of Surgery, Hippokrateion General Hospital of Athens, National and Kapodistrian University of Athens, 11527 Athens, Greece; antheo300@gmail.com (A.T.); spyros.smparounis@gmail.com (S.S.); 2 Department of Gastroenterology, “Konstantopouleio-Patision” General Hospital of Nea Ionia, 14233 Athens, Greece; panagaki.antonia@gmail.com; 3 Department of Pathology, Hippokrateion General Hospital of Athens, 11527 Athens, Greece; sofritsy@yahoo.gr (S.R.); eleni_st@windowslive.com (E.S.); chryssa.g@hotmail.com (C.G.); 4 Department of Medical Oncology, Hippokrateion General Hospital of Athens, 11527 Athens, Greece; mtheochari@gmail.com

**Keywords:** gastric cancer, tumor-infiltrating lymphocytes, lymph nodes, immune response, regression, chemotherapy, neoadjuvant, FLOT

## Abstract

**Background/Objectives**: Adenocarcinomas of the esophagogastric and gastric areas are often managed with a multimodal treatment including neoadjuvant chemotherapy and surgery. The impact of neoadjuvant chemotherapy on the host’s antitumoral immune response remains largely unknown. **Methods**: A retrospective review of a single-institution cohort of patients with adenocarcinoma of the stomach or esophagogastric area undergoing curative intent surgery after neoadjuvant chemotherapy FLOT (Fluorouracil, Leucovorin, Oxaliplatin, Docetaxel) was reviewed. After institutional ethics approval, pathologic slides were re-reviewed and tumor-infiltrating lymphocyte scores were calculated. Tumor-infiltrating lymphocytes (TILs) were studied in conjunction with tumor regression scores (TRG) and the degree of regression in the involved lymph nodes as well as in correlation with the lymph node ratio (the ratio of involved lymph nodes over the total number of lymph nodes resected). **Results**: A total of 106 patients were reviewed. No statistical correlation could be established between the tumor-infiltrating lymphocyte scores and the degree of regression in the primary tumor as well as with the partial response to chemotherapy of pathologically involved lymph nodes. The TIL score also did not correlate with the lymph node ratio in our patient cohort. A strong correlation was noted between TILs and lymph nodes that completely regressed after neoadjuvant chemotherapy. **Conclusions**: Tumor-infiltrating lymphocytes do not correlate with the response of the primary tumor or the partial response of the involved lymph nodes, but only with the complete response to neoadjuvant chemotherapy of tumor-involved lymph nodes. Our study focuses on the effects of neoadjuvant chemotherapy on tumor-infiltrating lymphocytes compared to the effects on the primary tumor and the involved lymph nodes.

## Simple Summary

Adenocarcinomas of the esophagogastric and gastric areas represent complex entities. Their management currently entails an increasing use of neoadjuvant therapies (mainly chemotherapy in Europe) followed by radical surgery. The effect that neoadjuvant treatments inflict on the anti-tumoral response of the host’s immune system remains largely unknown. We hereby report results from a single center on tumor-infiltrating lymphocytes assessed on surgical resection specimens after curative intent surgery in patients with adenocarcinomas of the stomach or esophagogastric junction areas. Our results suggest that tumor-infiltrating lymphocytes do not correlate with the tumor regression in the primary tumor but do correlate with the regression of the disease in the affected lymph nodes.

## 1. Introduction

The complex interplay between the immune system and adenocarcinomas of the stomach and esophago-gastric junction area is increasingly being recognized as key in understanding the intricate biologic processes involved in this disease entity. A recent publication [1] from two high-volume centers for surgically resected gastric adenocarcinomas undergoing adjuvant chemotherapy suggested that patients with a high density of tumor-infiltrating lymphocytes had better disease-free survival than their low-density counterparts. Most interestingly, patients in the low-density group appeared to benefit from adjuvant chemotherapy, thus suggesting that tumor-infiltrating lymphocytes might enable more accurate patient stratification into treatment groups.

In another report [2], high levels tumor-infiltrating lymphocytes that are CD3 positive appear to correlate with improved survival in gastric cancer patients undergoing curative intent surgery and seem to correlate with Epstein–Barr virus (EBV) positivity and programmed-death-ligand 1 (PD-L1) positivity. A recently published meta-analysis suggested that a high-density infiltrate of CD 3(+), CD 4(+), and CD 8(+) T cells correlates with improved survival while FOXP3(+) [forkheadboxprotein 3 positive] T cell subsets are not related to a prognosis in gastric cancer patients [3]. Analyzing the tumor–tumor microenvironment interactions from a spatially oriented point of view, Stein et al. [4] have suggested that it is the high intra-tumoral but not peritumoral inflammatory host response that is associated with a better prognosis. In another report with concordant conclusions from the authors [5], a high T-cell infiltration in the center of the tumor was associated with significant survival benefits, while the T-cell infiltration in the tumoral invasive front seemed to not correlate with overall survival.

An ever-increasing number of esophageal and gastric adenocarcinomas receive chemotherapy or chemoradiotherapy as a neoadjuvant treatment [6]. The response of pathologic lymph nodes after neoadjuvant chemotherapy has been postulated to be of significant prognostic value in predicting recurrence and survival in esophageal adenocarcinoma as suggested by a multicenter study from the UK [7]. In this report, a significant proportion of patients (more than 20%) demonstrated a discordant response between the degree of regression in the primary tumor and the pathologic lymph nodes. Further elaborating on the matter, pre-treatment tumor-infiltrating T cells influence the response to neoadjuvant chemoradiotherapy in esophageal adenocarcinoma [8], with a cytotoxic Th1 T-cell profile favoring a complete response to neoadjuvant treatment and the Th2 immunosuppressive T-cell profile being strongly associated with a non-response to neoadjuvant treatment. Differential expressions of various genes in tumor-infiltrating lymphocytes in gastric cancer patients have been recently described, thus suggesting distinct molecular signatures for patients with a high and low tumor lymphocyte infiltrate [9]. In this report, the authors created an elastic model with 11 gene signatures to predict the high-TIL subtype. Furthermore, other factors, such as cancer-associated fibroblasts (CAFs), have been postulated to regulate the tumor microenvironment and immune system evasion of cancer cells [10]. The infiltration of the tumor stroma by immune cells is a process that is not yet fully understood, with some determinants of the process such as mechanistic target of rapamycin(mTOR) only having been recently described [11].

We hereby report on a single-institution cohort of resected adenocarcinomas of the gastric and gastroesophageal junction area that underwent either gastrectomy or esophagectomy after neoadjuvant chemotherapy, with the focus on tumor-infiltrating lymphocytes and tumor regression status. We have attempted to elucidate the response of TILs to neoadjuvant chemotherapy in relation to the response of the primary tumor and involved lymph nodes.

## 2. Methods

Retrospective analyses of a prospectively maintained database of patients with resected adenocarcinomas of the stomach and esophagus (esophagogastric junction area) after neoadjuvant chemotherapy were reviewed.

Review of pathology reports was undertaken by a single consultant pathologist; thus, no inter-observer variability measurement was deemed necessary. Tumor-infiltrating lymphocyte scores were extracted from review of pathologic slides according to recommendations from the International TILs Working Group [12]. Tumor regression was reported according to the Mandard classification of tumor regression grades (TRGs) [13]. Values ranged from 1–5, with TRG 1 on one end demonstrating no tumor present and TRG 5 on the other end of the spectrum demonstrating no regression. When pathologic complete response (complete regression) was noted (TRG 1 according to Mandard patients), by definition, no tumor-infiltrating lymphocyte score was calculated. A numeric score with values between 0–100 was calculated. Based on some of our previous work [14], a cutoff value of 30 was chosen for designation of TIL-low and TIL-high patient groups. Regarding the selection of cut-off of 30 for the low group, the high group was established so that both groups have a number of more than 20 patients to be eligible for statistical analysis. To our knowledge, no established cut-off for high versus low TIL group exists in the literature. Pathologic review focused on number of lymph nodes harvested, looking at positive lymph nodes as well as negative lymph nodes. In a more elaborate analysis, positive lymph nodes with regression characteristics (partial nodal regression) and negative lymph nodes with regression characteristics (complete nodal regression) were also recorded in detail. Lymph node ratios (ratio of positive lymph nodes over total number of lymph nodes) were calculated for patients so as to factor in potential differences in number of lymph nodes harvested between different patients [15].

All statistical analyses were performed with the use of IBM* SPSS statistics program [Version 29.0.2.0 (20)] and Microsoft Excel for Mac OS (Version 16.94). Spearman’s Rho was used for all correlation analyses with *p*-values set for a 95% significance level.

The study received ethical approval from the Institutional Review Board of the Hippokrateion General Hospital of Athens (Decision Number: 16193/09-09-24). As this was a retrospective analysis with no patient intervention and with complete patient anonymization along the entire process, patients’ informed consent was waived.

## 3. Results

The results were available for 106 patients in total with 88 of them being assigned male at birth (83%) and 18 patients (17% of the total) being assigned female at birth. The mean age of the patients was 65 (the range was 32–82 years old). Sixty-four patients (64, 60.4%) underwent esophagectomy while the other forty-two patients underwent gastrectomy (42, 39.6%). The patients underwent Ivor Lewis esophagectomy, total gastrectomy or distal gastrectomy, depending on the tumor location and histologic characteristics with standard D2-level lymphadenectomy. All of the patients were diagnosed with adenocarcinomas of the esophagogastric junction or gastric area and all of them had received four cycles of neoadjuvant FLOT (fluorouracil, leucovorin, oxaliplatin, and docetaxel) chemotherapy according to institutional guidelines and after a multidisciplinary team meeting. The mean number of lymph nodes harvested was 29, ranging between 10 and59, while in only two patients, the lymph node count was less than 15 (11 and 13 lymph nodes, respectively).

When the patients with TRG 1 according to Mandard response were excluded from analysis, 88 patients from the original cohort remained for TIL score calculation. The TIL score was calculated for 83 out of 88 patients, as slides for pathologic analysis could not be retrieved, and thus the TIL score calculations of 5 patients were re-examined, although pathologic reports were available for all patients. Twenty-one (21) patients were designated as high-TIL versus sixty-two (62) that were designated as low-TIL using a TIL score cutoff of 30. The distribution of TIL scores according to TRG per Mandard are graphically depicted in Table 1.

When performing correlation analysis, the TRG according to Mandard status did not correlate with TIL groups (high or low), with a Spearman’s Rho value of 0.075 (*p* value 0.498). When correlating TIL groups with the lymph node ratio, again no statistical significance could be demonstrated for any correlation (Spearman’s Rho value −0.074; *p* value 0.507). To account for the potentially small sample size of patients, bootstrap analysis calculated at a sample of 1000 was undertaken which confirmed the results. Moreover, no statistically significant correlation could be demonstrated between TRG status and positive lymph nodes with regression characteristics (partial lymph node response), with a relevant Spearman’s Rho value of 0.090 (*p* value 0.406). Statistical significance was reached for the correlation between TRG status and negative lymph nodes with regression characteristics (complete lymph node response), with a Spearman’s Rho value of −0.261 (*p* value 0.015).

When analyzing the correlation between TIL groups and negative lymph nodes with regression characteristics (complete lymph node regression), a strong positive correlation was demonstrated with a Spearman’s Rho value of 0.237 and *p* value of 0.030. However, TIL groups classification did not correlate with positive lymph nodes with regression characteristics (partial lymph node regression) (*p* value 0.843).

When performing correlation analysis between TIL scores and lymph node ratios, we calculated a Spearman’s Rho value of minus 0.074, and a *p* value > 0.05, with the result not being statistically significant. Thus, no correlation could be proven between the magnitude of tumor-infiltrating lymphocytes and the degree of involvement of the tumor-draining lymphatic basin.

No long-term follow-ups on our patient cohort are available at the current time as results are still being collected; thus, survival analysis was not feasible, and the prognostic significance of our findings could not be explored. Moreover, the results regarding microsatellite instability status (MSI status) were not available for most patients.

## 4. Discussion

Tumor-infiltrating lymphocytes have been the focus of intense study in an attempt to elucidate cancer–host immune system interactions. A recent paper suggested that tumor-infiltrating lymphocytes interact not only with the primary tumor per se, but also with tumor deposits found in tumor-draining areas without any association with blood vessels or lymph nodes [16]. An increased burden of tumor deposits seems to be associated with a diminished number of TILs, implying a worse overall prognosis for patients. In other words, TILs present a barrier in the progression of cancer, whether this is in the form of the primary tumor or in the form of tumor deposits, which have long been associated with distant metastasis and worse overall survival [17].

The impact of neoadjuvant therapies on the host antitumoral immune response remains largely unknown. Our results suggest that the magnitude of infiltrating TILs (high or low) is not affected by the degree of tumoral response to neoadjuvant treatment (chemotherapy, in our case series study), suggesting that they present different biologic processes, despite demonstrating intricate interactions with each other. We have also demonstrated that the degree of tumor regression is mirrored in the regression status in lymph nodes, when only complete eradication of tumors from lymph nodes is demonstrated. More interestingly, when analyzing the correlation between TILs and complete regression in lymph nodes (negative nodes with a complete regression group), a strong statistically significant positive correlation was noted. The same could not be demonstrated, though, in the case of partial lymph node regression. These aforementioned results suggest that in patients demonstrating excellent response to neoadjuvant chemotherapy in involved lymph node stations, a significant tumor-associated lymphocytic infiltration is noted. The density of tumor-infiltrating lymphocytes, namely an increased density, has been associated with better overall survival outcomes in patients with gastric cancer [18]. The failure to demonstrate a correlation between the degree of tumor regression and the TIL score suggests that a high T-cell infiltration has no causal relationship with tumor regression in the primary tumor locus. However, a high T-cell infiltration seems to be a consistent finding in patients with involved lymph nodes that respond completely to neoadjuvant treatment. In our opinion, this finding reflects the distinct roles of tumor-infiltrating lymphocytes in the primary tumor locus and the tumor-draining lymph basin. Interestingly enough, tumor deposits are being increasingly recognized as key in cancer spread and metastasis and are being regarded as potentially more important than the lymphatic route of metastatic spread [19,20]. This might be a key finding to explain our findings, thus suggesting that if no visible tumor cells can be identified in the previously involved tumor lymphatic basin, then from a teleological point of view, the immune response that has been mounted is strong enough to completely eradicate potentially not only tumor cells in the involved lymph nodes, but also the aggressive phenotype subpopulation of tumor deposits that reside nearby. This might be particularly the case if we consider that tumor deposits are usually identified by pathologists in neoadjuvant-therapy-naïve patients, and that they are sometimes hard to distinguish even from extranodal tumor invasion [21]. Therefore, in pathologic examination of resected specimens after neoadjuvant therapies, deciphering lymph nodes with complete regression from tumor deposits that have completely been eradicated might be even more complicated. From this point of view, the finding that tumor-infiltrating lymphocytes do not correlate with the primary tumor degree of response to treatment can also be explained, because tumor-infiltrating lymphocytes, by definition, are a clinical entity that is not to be used in the case of complete primary tumor response due to the mere absence of a viable tumor and therefore tumor-associated lymphatic cells.

When we examined the lymph node ratio to the TIL correlation in our patient cohort, no statistically significant correlation could be demonstrated. In our opinion, this highlights that the TIL response should not be viewed from a quantitative point of view but rather from a qualitative perspective. When considering that the lymph node ratio reflects the extent that lymph nodes have been overwhelmed by cancer spread, then failure to correlate the TIL score with the lymph node ratio implies that the tumor-associated lymphocytic response is a biologic process distinct from the degree of tumoral lymphatic spread, which we often quantify as the nodal (N) stage in most cancer-staging systems. This might have important implications for the staging systems of gastric and gastroesophageal junction adenocarcinomas.

Our study suffers from a significant number of limitations. Although being prospectively collected, our data have been retrospectively reviewed on a single-institution basis and are therefore subject to selection bias. The lack of available data on MSI status significantly restricts the generalizability of our conclusions, as the exact proportion of high-MSI patients in our cohort remains unknown. Another limitation is the lack of long-term follow-up to enable analysis of our results to potentially identify their prognostic value as well as our inability to provide qualitative data on the kind (namely molecular markers) of tumor-infiltrating cells in our study. In a recent systematic review and meta-analysis on tumor-infiltrating lymphocytes in gastric cancer [3], tumor cell infiltration with CD 3(+) tumor-infiltrating lymphocytes was associated with a favorable survival effect. In the same analysis, when analyzing the effect of tumor infiltration with “regulatory” T cells [expressed as FOXP3(+) T cells], a negative effect on overall survival was noted. These results highlight that a tumor-associated T-cell infiltration should not only be assessed from a quantitative perspective (present/absent and TIL score) but also from a qualitative point of view. A T-cell infiltration expressing pro- or counter-tumoral properties has been well documented in other malignancies of the gastrointestinal tract, as in colorectal cancer [22], where opposing functions of distinct regulatory T cells have been described. To add a further degree of complexity, neoadjuvant chemotherapy affects not only the tumor and associated lymph nodes but also the host’s immune response. A study by Jomrich. G. et al. [23] demonstrated that neoadjuvant chemotherapy significantly decreased the expression of Programmed Death Ligand 1 (PD-L1) in cancer cells. It is, thus, reasonable to assume that neoadjuvant chemotherapy might diminish a potential benefit from PD-L1 inhibitors in the adjuvant setting for patients who would have been candidates for immunotherapy in the neoadjuvant setting. Further strengthening this assumption, Tanaka et al. [24] suggested a harmful effect of PD-L1 expression in esophageal cancer patients receiving neoadjuvant chemotherapy that was not also noted in patients not receiving neoadjuvant chemotherapy. Although the lack of PD1/PD-L1 expression in our patients is a potential limitation of our study, our focus of study has been tumor-infiltrating lymphocytes in patients receiving neoadjuvant chemotherapy. A PD1/PD-L1-positive status would probably have precluded these patients from receiving neoadjuvant chemotherapy, given the increasing data indicating that they benefit little from it. However, it would be enlightening to study the disease regression in lymph nodes versus the primary tumor and stratify patients according to PD-1/PD-L1 status. Hopefully, in the near future, data from the long-term follow-up shall be available for most of our patients, thus enabling survival analysis and the development of prognostic models, along with more data on the surface markers expressed as well as molecular characterization of tumor-infiltrating lymphocytes. Another potential limitation of our study is the lack of immunohistochemical profiling of tumor-infiltrating lymphocytes. We have instead provided data of a quantitative rather than qualitative nature on the immune infiltration in our patient cohort. We have purposefully done so in an attempt to provide an easily reproducible method for estimating the immune infiltration in adenocarcinomas of the gastroesophageal junction/gastric area, rather than engaging in profiling of TILs, which has not been standardized across the various studies in the literature.

Tumor-infiltrating lymphocytes might be a key pathologic parameter to better understand tumor–immune system interactions and better inform treatment decisions, as we are only now beginning to understand these complex interactions. A recently reported phase II trial [25] on neoadjuvant immunotherapy in high-microsatellite (MSI-H) adenocarcinomas of the esophagogastric junction and gastric areas reported pathologic complete response rates of 60% and major pathologic response rates of 80% with excellent progression-free and overall survival at the 24-month time mark. In this trial, the authors even propose an organ preservation approach obviating the need for surgery in excellent responders who are put on close surveillance in a method analogous to the watch-and-wait method for rectal cancer patients [26]. Our results suggest that tumor-infiltrating lymphocytes do not correlate with the degree of tumoral regression or the partial regression of the involved lymph nodes except in the case of complete regression of a previously involved lymph node. It is perhaps the case that a strong T-cell infiltration after neoadjuvant chemotherapy (which downsizes tumoral PD-L1 expression and hampers the anti-tumoral T cell response) might therefore suggest from a teleologic point of view that despite the negative effect of chemotherapy on the antitumoral immune response, a robust response is still elicited that can turn previously positive lymph nodes into lymph nodes with complete tumoral regression. However, even in responders after neoadjuvant immunotherapy, this response cannot be clinically assessed by means of endoscopy or Positron Emission (PET) imaging, as suggested by a recent report, in which the authors described persistent ulceration as well as a decrease in standardized uptake volumes (SUV) even in patients demonstrating a pathologic complete response subsequently during surgical operation [27]. Long-term results regarding the combination of chemotherapy with immunotherapy in the neoadjuvant setting are awaited from numerous studies [28,29,30]. Interestingly enough, immunotherapy in the adjuvant setting in gastric/gastroesophageal junction adenocarcinoma patients with persisting lymph node positivity on surgical specimens after surgery or incomplete (R1) resections following neoadjuvant chemotherapy also failed to show any benefit and was in fact associated with worse outcomes [31], which aligns with our findings that TILs do not correlate with persistently positive lymph nodes after neoadjuvant chemotherapy.

## 5. Conclusions

We have demonstrated in a case series study from a prospectively based database on gastric and gastroesophageal junction patients that tumor-infiltrating lymphocytes do not correlate with the degree of tumor regression after neoadjuvant chemotherapy but do correlate with complete regression in the tumor-associated involved lymph nodes. Our study suffers from significant limitations, such as its retrospective nature, lack of follow-up and lack of molecular-based data on the patients. Hopefully, in the near future, immunohistochemical studies on the CD3, CD4, CD8 and Treg markers performed in our patient cohort might further elucidate the biology of tumor-infiltrating lymphocytes. We are currently prospectively following patients from our institution; thus, survival data shall become available at a future time. More studies are needed to further elucidate the complex interactions between tumor-infiltrating cells and gastric/gastroesophageal junction adenocarcinomas, especially after neoadjuvant treatments. In our opinion, further characterizing tumor-infiltrating lymphocytic responses might be a key step in harnessing the power of immunotherapy in a more effective and more precise fashion.

## Figures and Tables

**Table 1:** TIL scores across TRG Mandard stages.

TILs Subgroups Across TRG per Mandard Stages						
Count						
		TRG Mandard				Total Number of Patients
		2	3	4	5	
TIL subgroups	No available path data	2	0	2	1	5
	TILs high	5	5	7	4	21
	TILs low	13	24	18	7	62
Total number of patients		20	29	27	12	88

## Data Availability

The original contributions presented in this study are included in the article. Further inquiries can be directed to the corresponding authors.

## References

[1] Liu D.H., Kim Y.W., Sefcovicova N., Laye J.P., Hewitt L.C., Irvine A.F., Vromen V., Janssen Y., Davarzani N., Fazzi G.E. (2023). Tumour infiltrating lymphocytes and survival after adjuvant chemotherapy in patients with gastric cancer: Post-hoc analysis of the CLASSIC trial. Br. J. Cancer.

[2] Pereira M.A., Ramos M.F., Cardili L., de Moraes R.D., Dias A.R., Szor D.J., Zilberstein B., Alves V.A., de Mello E.S., Ribeiro U. (2024). Prognostic implications of tumor-infiltrating lymphocytes within the tumor microenvironment in gastric cancer. J. Gastrointest. Surg..

[3] Cao X., Kang Y., Tai P., Zhang P., Lin X., Xu F., Nie Z., He B. (2024). Prognostic role of tumor-infiltrating lymphocytes in gastric cancer: A systematic review and meta-analysis. Clin. Res. Hepatol. Gastroenterol..

[4] Stein A.V., Dislich B., Blank A., Guldener L., Kröll D., Seiler C.A., Langer R. (2017). High intratumoural but not peritumoural inflammatory host response is associated with better prognosis in primary resected oesophageal adenocarcinomas. Pathology.

[5] Schoemmel M., Loeser H., Kraemer M., Wagener-Ryczek S., Hillmer A., Bruns C., Thelen M., Schröder W., Zander T., Lechner A. (2021). Distribution of tumor-infiltrating-T-lymphocytes and possible tumor-escape mechanisms avoiding immune cell attack in locally advanced adenocarcinomas of the esophagus. Clin. Transl. Oncol..

[6] Lombardi P.M., Pansa A., Basato S., Giorgi L., Perano V., Marano S., Castoro C. (2023). Facing adenocarcinoma of distal esophagus and esophagogastric junction: A CROSS versus FLOT propensity score-matched analysis of oncological outcomes in a high-volume institution. Updates Surg..

[7] Moore J.L., Green M., Santaolalla A., Deere H., Evans R.P.T., Elshafie M., Lavery A., McManus D.T., McGuigan A., Douglas R. (2023). Pathologic Lymph Node Regression After Neoadjuvant Chemotherapy Predicts Recurrence and Survival in Esophageal Adenocarcinoma: A Multicenter Study in the United Kingdom. J. Clin. Oncol..

[8] Goedegebuure R.S.A., Harrasser M., de Klerk L.K., van Schooten T.S., van Grieken N.C.T., Eken M., Grifhorst M.S., Pocorni N., Jordanova E.S., van Berge Henegouwen M.I. (2021). Pre-treatment tumor-infiltrating T cells influence response to neoadjuvant chemoradiotherapy in esophageal adenocarcinoma. Oncoimmunology.

[9] Yu W., Wang S., Rong Q., Ajayi O.E., Hu K., Wu Q. (2022). Profiling the Tumor-Infiltrating Lymphocytes in Gastric Cancer Reveals Its Implication in the Prognosis. Genes.

[10] Mak T.K., Li X., Huang H., Wu K., Huang Z., He Y., Zhang C. (2022). The cancer-associated fibroblast-related signature predicts prognosis and indicates immune microenvironment infiltration in gastric cancer. Front. Immunol..

[11] Nakazawa N., Sohda M., Katayama A., Ide M., Shimoda Y., Tateno K., Watanabe T., Sano A., Sakai M., Yokobori T. (2023). Infiltration of Gastric Cancer Stroma by Tumor-Infiltrating Lymphocytes Correlates with Mechanistic Target of Rapamycin Signaling. Oncology.

[12] Salgado R., Denkert C., Demaria S., Sirtaine N., Klauschen F., Pruneri G., Wienert S., Van den Eynden G., Baehner F.L., Pénault-Llorca F. (2015). The evaluation of tumor-infiltrating lymphocytes (TILs) in breast cancer: Recommendations by an International TILs Working Group 2014. Ann. Oncol..

[13] Henckens S.P.G., Liu D., Gisbertz S.S., Kalff M.C., Anderegg M.C.J., Crull D., Daams F., van Dalsen A.D., Dekker J.W.T., van Det M.J. (2024). Prognostic value of Mandard score and nodal status for recurrence patterns and survival after multimodal treatment of oesophageal adenocarcinoma. Br. J. Surg..

[14] Seretis F., Glava C., Smparounis S., Riga D., Karantzikos G., Theochari M., Theodorou D., Triantafyllou T. (2024). Tumor-Infiltrating Lymphocytes in Resected Esophageal and Gastric Adenocarcinomas Do Not Correlate with Tumor Regression Score After Neoadjuvant Chemotherapy: Results of a Case-Series Study. Cancers.

[15] Jannasch O., Schwanz M., Otto R., Mik M., Lippert H., Mroczkowski P. (2025). Lymph Node Yield and Lymph Node Ratio for Prognosis of Long-Term Survival in Gastric Carcinoma. Cancers.

[16] Li X., Yang J. (2022). Association of tumor deposits with tumor-infiltrating lymphocytes and prognosis in gastric cancer. World J. Surg. Oncol..

[17] Wenquan L., Yuhua L., Jianxin C., Hongqing X., Kecheng Z., Jiyang L., Yunhe G., Yi L., Wang Z., Shaoqing L. (2020). Tumor deposit serves as a prognostic marker in gastric cancer: A propensity score-matched analysis comparing survival outcomes. Cancer Med..

[18] Yu P.C., Long D., Liao C.C., Zhang S. (2018). Association between density of tumor-infiltrating lymphocytes and prognoses of patients with gastric cancer. Medicine.

[19] Graham Martínez C., Knijn N., Verheij M., Nagtegaal I.D., van der Post R.S. (2019). Tumour deposits are a significant prognostic factor in gastric cancer—A systematic review and meta-analysis. Histopathology.

[20] Lee H.S., Lee H.E., Yang H.K., Kim W.H. (2013). Perigastric tumor deposits in primary gastric cancer: Implications for patient prognosis and staging. Ann. Surg. Oncol..

[21] Etoh T., Sasako M., Ishikawa K., Katai H., Sano T., Shimoda T. (2006). Extranodal metastasis is an indicator of poor prognosis in patients with gastric carcinoma. Br. J. Surg..

[22] Huang X., Feng D., Mitra S., Andretta E.S., Hooshdaran N.B., Ghelani A.P., Wang E.Y., Frost J.N., Lawless V., Vancheswaran A. (2025). Opposing Functions of Distinct Regulatory T Cell Subsets in Colorectal Cancer. bioRxiv.

[23] Jomrich G., Kollmann D., Ramazanova D., Ristl R., Grose R.P., Ilhan-Mutlu A., Preusser M., Fassnacht C., Tsai Y.C., Guenova E. (2022). Expression of programmed cell death protein 1 (PD-1) and programmed cell death 1 ligand (PD-L1) in adenocarcinomas of the gastroesophageal junction change significantly after neoadjuvant treatment. Eur. J. Surg. Oncol..

[24] Tanaka K., Miyata H., Sugimura K., Kanemura T., Hamada-Uematsu M., Mizote Y., Yamasaki M., Wada H., Nakajima K., Takiguchi S. (2016). Negative influence of programmed death-1-ligands on the survival of esophageal cancer patients treated with chemotherapy. Cancer Sci..

[25] Raimondi A., Lonardi S., Murgioni S., Cardellino G.G., Tamberi S., Strippoli A., Palermo F., De Manzoni G., Bencivenga M., Bittoni A. (2025). Tremelimumab and durvalumab as neoadjuvant or non-operative management strategy of patients with microsatellite instability-high resectable gastric or gastroesophageal junction adenocarcinoma: The INFINITY study by GONO. Ann. Oncol..

[26] Giunta E.F., Bregni G., Pretta A., Deleporte A., Liberale G., Bali A.M., Moretti L., Troiani T., Ciardiello F., Hendlisz A. (2021). Total neoadjuvant therapy for rectal cancer: Making sense of the results from the RAPIDO and PRODIGE 23 trials. Cancer Treat. Rev..

[27] Shannon A.B., Mehta R., Mok S.R., Lauwers G.Y., Baldonado J.J.A.R., Fontaine J., Pimiento J.M., Sinnamon A.J. (2024). Clinical and Pathologic Response to Neoadjuvant Immunotherapy in DNA Mismatch Repair Protein-Deficient Gastroesophageal Cancers. Ann. Surg. Oncol..

[28] Long B., Zhou H., Yu Z., Zhu J., Yang H., Huang Z., Wei D., Chen S., Yang X., Zhao X. (2025). Neoadjuvant cadonilimab plus FLOT chemotherapy in locally advanced gastric/gastroesophageal junction adenocarcinoma: A multicenter, phase 2 study. Med.

[29] Lin G.T., Yan C., Li L.J., Qiu X.W., Zhao Y.X., Lin J.L., Chen Y.J., Feng C., Chen S.Q., Xie J.W. (2025). Combining Apatinib and Oxaliplatin Remodels the Immunosuppressive Tumor Microenvironment and Sensitizes Desert-Type Gastric Cancer to Immunotherapy. Cancer Res..

[30] Li Y., Cao Y., Wang X., Li C., Zhao L., Li H. (2025). Effects of perioperative treatment of resectable adenocarcinoma of esophagogastric junction by immunotherapy (Adebrelimab) combined with chemotherapy (XELOX): Protocol for a single-center, open-labeled study (AEGIS trial, neoadjuvant immunochemotherapy). BMC Cancer.

[31] Lordick F., Mauer M.E., Stocker G., Cella C.A., Ben-Aharon I., Piessen G., Wyrwicz L., Al-Haidari G., Fleitas-Kanonnikoff T., Boige V. (2025). Adjuvant immunotherapy in patients with resected gastric and oesophagogastric junction cancer following preoperative chemotherapy with high risk for recurrence (ypN+ and/or R1): European Organisation of Research and Treatment of Cancer (EORTC) 1707 VESTIGE study. Ann. Oncol..

